# Monitoring and evaluation of intervals from onset of fever to diagnosis before “1-3-7” approach in malaria elimination: a retrospective study in Shanxi Province, China from 2013 to 2018

**DOI:** 10.1186/s12936-019-2865-0

**Published:** 2019-07-12

**Authors:** Ting Wang, Shui-Sen Zhou, Jun Feng, Myo Minn Oo, Jing Chen, Chang-Fu Yan, Yi Zhang, Ping Tie

**Affiliations:** 1 Shanxi Center for Disease Control and Prevention, Taiyuan, 030012 China; 2 National Institute of Parasitic Diseases, Chinese Center for Disease Control and Prevention, Shanghai, 200025 China; 3 Center for Operational Research, International Union Against Tuberculosis and Lung Disease, Mandalay, 05021 Myanmar; 4 National Institute for Viral Disease Control and Prevention, Chinese Center for Disease Control and Prevention, Beijing, 102206 China

**Keywords:** Malaria, Shanxi, Case diagnosis, Elimination

## Abstract

**Background:**

China’s 1-3-7 approach was extensively implemented to monitor the timeframe of case reporting, case investigation and foci response in the malaria elimination. However, activities before diagnosis and reporting (before ‘1’) would counteract the efficiency of 1-3-7 approach but few data have evaluated this issue. This study aims to evaluate the timelines between onset of fever and diagnosis at healthcare facilities in Shanxi Province.

**Methods:**

Routine data were extracted from IDIRMS and NMISM database from 2013 to 2018. Time intervals between onset of fever and healthcare-seeking and between healthcare-seeking and diagnosis were calculated. Each of the documented malaria cases was geo-coded and paired to the county-level layers of polygon.

**Results:**

A total of 90 cases were reported in 2013–2018 in Shanxi Province, and 73% of cases reported at provincial health facilities. All malaria cases were imported from Africa (90%) and Southeast Asia (10%) especially around the Chinese Spring Festival (n = 46, 51%). The median days between fever and healthcare-seeking and between healthcare-seeking and diagnosis of malaria were 3 and 2, respectively.

**Conclusions:**

The current “1-3-7” approach is well executed in Shanxi Province, but delays intervals observed in case finding before 1-3-7 approach occurred in all levels of facilities in Shanxi Province, which imply that more efforts are highlighted for timely case finding. Health education should be provided for improving awareness of healthcare-seeking, and various technical training aiming at the physicians should be carried out to improve diagnosis of malaria.

## Background

Malaria, a globally transmitted disease, is more commonly endemic in tropical and subtropical regions, and remains as one of the most important parasitic diseases. In 2017, estimated 219 million malaria cases and 435,000 deaths were reported worldwide, and 70% of them occurred in 11 countries [1]. China historically reported a high incidence of malaria in a wide range of geographic areas in last 1960s and 1970s, *Plasmodium vivax* and *Plasmodiumfalciparum* were dominant in the southern regions [2–4]. Fortunately, great achievements have been made in controlling local transmission over several decades, and no indigenous case was reported nationwide for the first time in 2017 [1, 5].

Shanxi Province is one of historically malaria-endemic regions in China with three large outbreaks in 1950, 1970, and 1974 [6, 7], respectively. After decades of efforts for malaria control and prevention, no indigenous cases reported till 2012 [6, 8]. Recently, with the increasing of international communication under the "Belt and Road Initiative” [9], people involved in global commercial activities also has been increasing, which also led to an increase of imported cases in Shanxi [10, 11].

Therefore, malaria prevention and control is of particular importance in overseas populations who travel/work within in China. ‘1-3-7’ approach, which refers to three steps: (1) case reporting within 24 h; (2) case confirmation and investigation within 3 days; (3) and the foci investigation and response within 7 days, was crucial to rapid detection of every malaria infection and rapid and appropriate treatment to identified cases [5, 12–14]. Previous studies in China primarily focused on the ‘1-3-7’ approach, while few studies focused on time interval before the step ‘1’. Most of reported malaria cases in Shanxi were *P.falciparum*, which has a shorter incubation period and rapid onset than *P. vivax*, so it is quite critical for potential cases to seek to the health facility to obtain timely diagnosis and treatment. However, the time delay from onset of fever to diagnosis could increase case fatality of acute febrile illnesses. Correspondingly, the detailed issues on the time interval before the step ‘1’ attracted the interest as it implies the patient's awareness of healthcare-seeking and the timeliness and accuracy of diagnosis by health facilities. Herein, the study aims to analyse and evaluate the time intervals from onset of fever symptom to final case diagnosis at different levels of healthcare facilities in Shanxi Province from 2013–2018.

## Methods

### Study design

This is a retrospective cross-sectional study using monitoring data for infectious diseases of Shanxi Province, China [12, 15] between 2013 and 2018. The details of this system have also been described previously [13].

### Setting

Shanxi Province has current 11 administrative regions, 119 counties and 1398 townships, and it also includes 4250 health facilities (including clinics), 131 centres for disease control and prevention (CDC), and 135 maternity and child care centres [16].

### Malaria case definition

A malaria case is defined to be positive infection of parasitaemia by quality controlled laboratory diagnosis regardless of clinical symptoms. An imported malaria case refers to persons who had acquired was infection outside of local areas or China [2]. In accordance to the four-catalogue classification setting by the National Malaria Elimination Action Plan [17], all counties in Shanxi province and its corresponding strategy is to strengthen surveillance on the imported potential people to prevent from re-establishment [2].

Once a malaria case is reported to the system, the staff from CDC will further confirm whether the case is indigenous or imported. A blood smear and a blood-spot filter paper sample are collected and are detected by microscopy and polymerase chain reaction (PCR) in the provincial qualified laboratory [18, 19]. Case are classified by a travel history, and the patient may be (1) indigenous; (2) imported; (3) introduced; (4) induced; or (5) unknown.

### Data source and variables

Data were extracted from the National Infectious Diseases Information Reporting Management System (IDIRMS). Main information includes date of onset of fever, date of first healthcare-seeking, date of malaria diagnosis, some sociodemographic information can also be collected, including age at diagnosis, gender, occupation, levels of health-care facility, *Plasmodium* species, country.

### Statistical analysis

Categorical data was summarized using frequency and proportion, and continuous variable was summarized by medians and interquartile range (IQR). Time intervals between onset of fever and healthcare-seeking, and interval between healthcare-seeking and diagnosis was calculated. Data were analysed using SPSS software (Version 13, IBM Corp, Armonk, NY, USA). Each of the documented malaria cases was geo-coded and paired to the county-level layers of polygon and pointed with ArcGIS 10.1 software (Environmental Systems Research Institute, Inc, Redlands, CA, USA).

### Ethics approval

This study has been approved by the Ethics Review Board of the Shanxi CDC. All data from the surveillance system were in secondary data, no informed consent could be obtained.

## Results

### Malaria in Shanxi Province between 2013 and 2018

A total of 90 cases of malaria were reported from January 2013 to September 2018, among which 95% (85) were male; 46% (42) were aged 30–50 years, and 38% (34) were less than 30 years; 72% (65) were outdoor workers (Table 1).

**Table 1:** Epidemiological characteristics of malaria cases and the interval between fever onset and healthcare-seeking, between healthcare-seeking and diagnosis of cases in Shanxi Province, China from 1st January 2013 to 30th September 2018

Demographic characteristics	Sub-category	Number	%	Time taken to health-seeking^a^		Time taken to malaria diagnosis^b^	
				Median	(IQR)	Median	(IQR)
Total		90	100	3	(1–7)	2	(0–7)
Age (years)	≤ 30	34	38	2	(0–5)	2	(0–6)
	31–50	42	46	3	(0–7)	1	(0–7)
	≥ 51	14	16	6	(1–13)	3	(1–9)
Gender	Male	85	95	3	(1–7)	1	(0–6)
	Female	5	5	1	(0–2)	12	(3–13)
Occupation	Outdoor workers	65	72	3	(1–7)	2	(0–7)
	Indoor workers	25	28	3	(1–7)	1	(0–8)
Level of health facility reported malaria cases	County	7	8	1	(0–4)	4	(2–7)
	Prefecture	17	19	4	(0–7)	3	(1–8)
	Provincial	66	73	3	(1–7)	1	(0–7)
*Plasmodium* species	*P. malariae*	2	2	34	(30–37)	5	(3–8)
	*P. ovale*	5	5	7	(2–13)	2	(0–7)
	*P. vivax*	15	17	5	(1–8)	2	(1–6)
	*P. falciparum*	64	71	2	(0–5)	2	(0–7)
	Mixed-infection	4	5	5	(3–7)	0	(0–0)
Origin continent	Southeast Asia	9	10	7	(5–8)	2	(0–7)
	Africa	81	90	2	(0–6)	1	(0–7)

^a^Measured from onset of fever to healthcare-seeking

^b^Measured from healthcare-seeking to malaria diagnosis

**Fig. 1:**
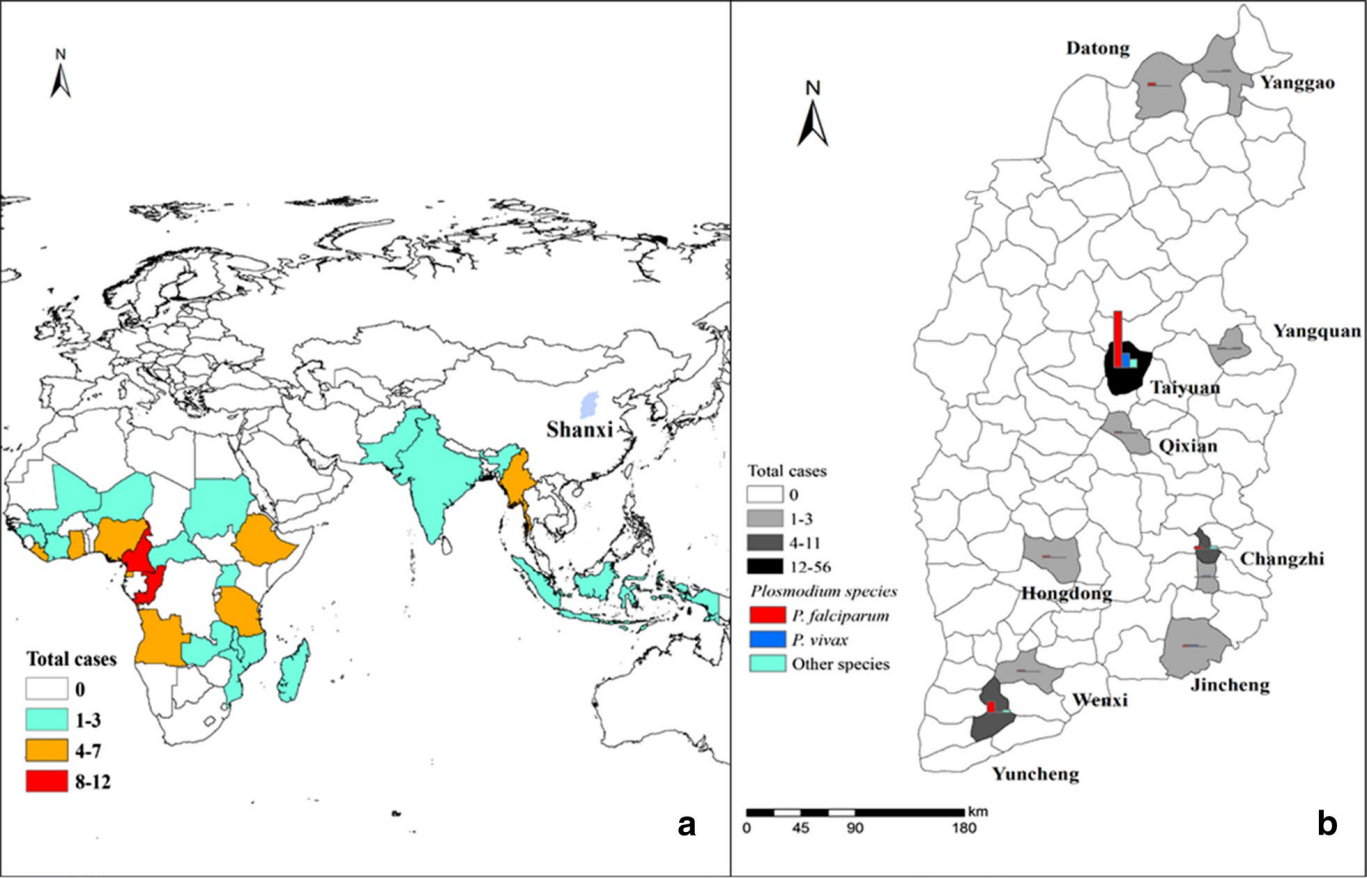
The number of the origin of imported cases (**a**) and distribution of imported cases in Shanxi Province of China stratified by species (**b**) between 1th January 2013 and 31th December 2018

**Fig. 2:**
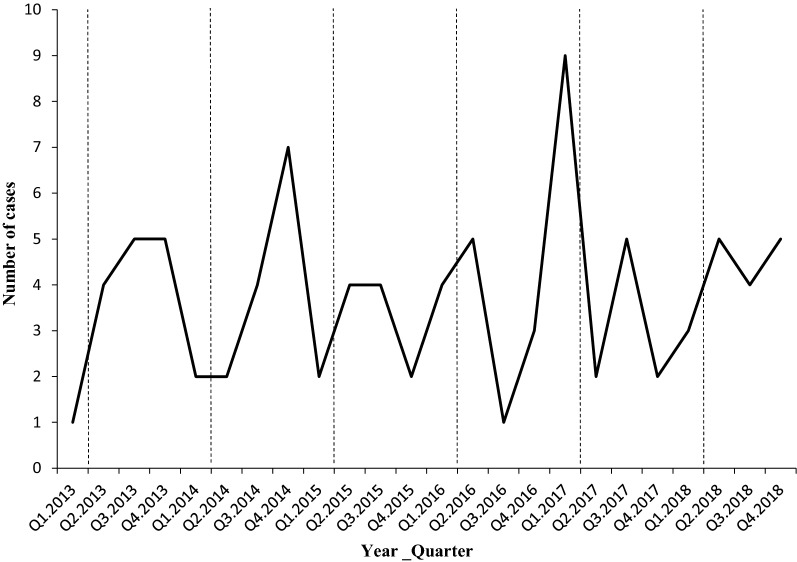
Quarter distribution of malaria cases reported in Shanxi Province, China between 1th January 2013 and 31th December 2018. *The dotted line in the figure indicates the Spring Festival time

All cases were reported from 15 counties of 8 prefectures (Fig. 1), 57 (63%) cases were detected in the Yingze District of Taiyuan, the provincial capital of Shanxi, and 11 (13%) cases were from the Salt Lake District of southern Shanxi. All cases were imported from 21 countries in Africa (90%) or 5 countries in Southeast Asia (10%). More cases were imported from three countries, 12 (13%) cases from the Republic of the Congo, 12 (13%) cases from Cameroon, and 8 (9%) cases from Nigeria. Cases were more frequent to be reported in each late January (23%) or early February (28%), lunar last month or first month of each year when most of oversea workers returned home for celebrating traditional Chinese Spring Festival (Fig. 2).

**Fig. 3:**
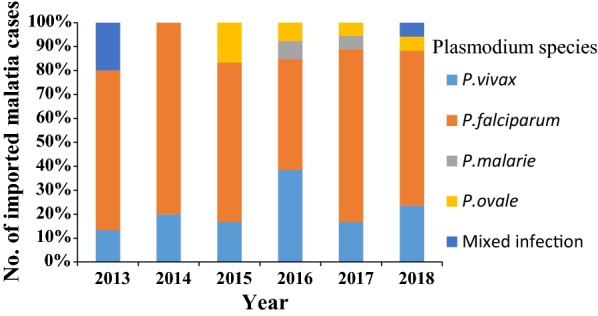
Malaria cases reported in Shanxi Province, China between 1th January 2013 and 31th December 2018

*Plasmodium* species were detected among malaria cases, most of cases had the infection of *P. falciparum* (71%, 64), followed by *P. vivax* (17%, 15) between 2013 and 2018, and the rest cases (12%, 11) were infected by *Plasmodium ovale* (5%), *Plasmodium malariae* (2%) and mixed infection cases (5%) between 2015 and 2018 because of utilization of PCR in CDC since 2015 (Fig. 3, Table 2).

**Table 2:** *Plasmodium* Species of Malaria in different countries of origin

	*P. falciparum*	*P. vivax*	*P. malariae*	*P. ovale*	Mixed-infection	Total
Republic of Congo	10	0	0	2	0	12
Cameroon	9	0	0	2	1	12
Nigeria	7	0	1	0	0	8
Equatorial Guinea	5	0	0	0	0	5
Ghana	4	0	0	0	0	4
Angola	4	0	0	0	0	4
Liberia	3	0	0	0	2	5
Mozambique	3	0	0	0	0	3
Ivory Coast	3	0	0	0	0	3
Uganda	2	0	0	0	0	2
Zambia	2	0	0	0	0	2
Tanzania	2	1	1	0	0	4
Malawi	2	0	0	0	0	2
Central African	2	0	0	0	0	2
Ethiopia	1	5	0	0	1	7
Mali	1	0	0	0	0	1
Madagascar	1	0	0	0	0	1
Niger	1	0	0	0	0	1
Guinea	1	0	0	0	0	1
Sierra Leone	1	0	0	0	0	1
Myanmar	0	4	0	0	0	4
Indonesia	0	1	0	0	0	1
Sudan	0	1	0	0	0	1
Pakistan	0	2	0	0	0	2
India	0	1	0	0	0	1
Brunei	0	0	0	1	0	1
Total	64	15	2	5	4	90

### Time interval between onset of fever and malaria diagnosis

**Fig. 4:**
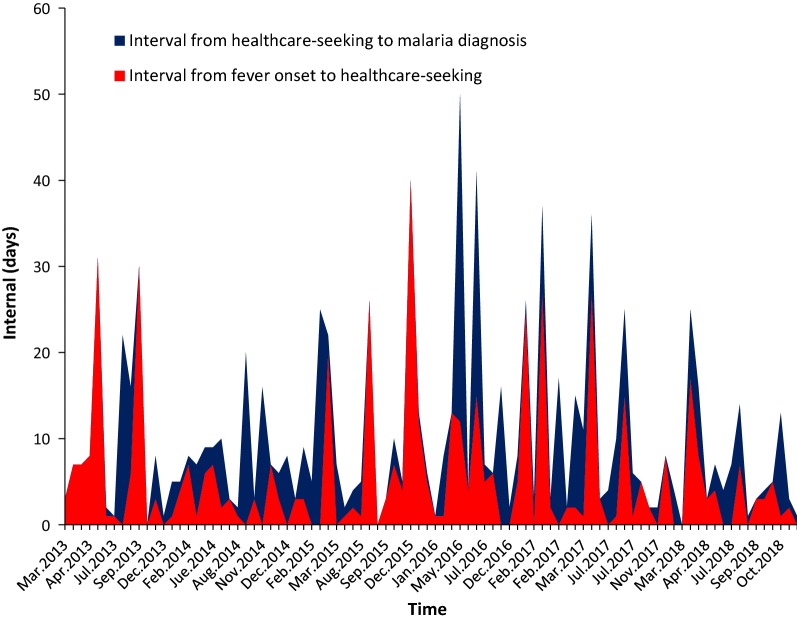
The interval from onset of fever to healthcare-seeking and the interval from healthcare-seeking to diagnosis among malaria cases in Shanxi province, China between 1th January 2013 and 31th December 2018

66 (73%) cases were diagnosed and reported by provincial qualified hospitals, 17 (19%) and 7 (8%) cases were found by prefecture hospitals and county facilities including hospitals and county CDC, respectively. The median time between onset of fever and healthcare-seeking was 3 days (IQR, 1–7 days); and median time between healthcare-seeking and malaria diagnosis was 2 days (IQR, 0–7 days) (Fig. 4). These two-time intervals across different health-care settings had no significant difference (P > 0.05).

## Discussion

The study found that time interval either between fever onset and health-seeking or between health-seeking and final diagnosis was long in Shanxi Province. Due to the globalization and integration, the imported malaria becomes a great challenge to the elimination programme in Shanxi Province similar as the whole country and other provinces [2, 20–26]. More patients in Shanxi Province are engaged in outdoor work than indoor work. Due to the launch of "One Belt, One Road" strategy, many large state-owned companies, such as Chinese railway construction, go to Africa to conduct infrastructure projects, such as Dams, railways and roads. However, most of the migrant workers were diagnosed with malaria abroad in private clinics and take anti-malarial drugs themselves instead of going to hospital after they came back from endemic areas [27]. In addition, the personnel's awareness for malaria diagnosis and treatment is low [28, 29], which also result in the poor health-seeking behaviour. On the other hand, health facilities at or below the county level lack experience in malaria diagnosis and treatment [2]. Many publications have pointed out that the malaria awareness rate of prevention and treatment among medical staff in primary health care institutions is low [10], which hardly to detect the *Plasmodium* parasites in the blood. Finally, poor microscopic examination capacity at the primary level in Shanxi also led to delay in malaria diagnosis and treatment [3, 27, 30–32], this is also observed in Jiangsu Province [33].

All the cases in Shanxi Province between 2013 and 2018 were diagnosed and reported by health facilities at or above the county level, and most of them were reported by provincial health facilities. This was because the Third People's Hospital, recognized as provincial designated infectious hospital, accounted for 52% of the total reported cases. The migrant workers were inclined to take health facility at or above county level as their first consultation [29]. On the other hand, the importance of the provincial designated infectious hospital is responsible for timely diagnosis and appropriate treatment for the malaria patients, sometimes also was set up as a sentinel hospital to carry out blood examinations of the febrile people, thus the capacity could be maintained [34].

*Plasmodium ovale* and *P. malariae* were first diagnosed in 2015 in Shanxi Province. This may due to the provincial reference laboratory, which was established in 2014, to use PCR and microscopy to re-confirm all positive and suspected cases sent by the county and prefecture CDC in Shanxi Province. Because of the tendency of *P. oval*e is capable of relapsing after treatment of the primary infection due to hypnozoite activity, and late relapse may occur because of the failure to receive or adhere to the prescribed chemoprophylaxis. Therefore, it is urgent for CDC staff to carry out radical cure before the next transmission season, otherwise, it may lead to activation of hypnozoites in the liver causing the relapse of infection after returning from the endemic areas [35].

Some documents were reported on time consumption from onset of fever to malaria diagnosis at different level of health facilities. To analysed the average time interval 1 day from onset of fever to healthcare-seeking and 3 days from healthcare-seeking to diagnosis of malaria [10]. To compare this, Shanxi has longer intervals from onset of fever to healthcare-seeking than the average level of the whole country. Deng et al*.* [36] reported that average the time spend by cases from onset of malaria to diagnosis was 5.1 days in Yunnan Province. Similarity, Feng et al*.* [12] reported that it took an average of 8.5 days for patients to get diagnosed from onset of fever along the China-Myanmar border. This was because the mobile and migrant population is frequent and no natural barrier existed in the border area, so the interval is longer than Shanxi Province. Other papers reported the average time from onset to diagnosis of malaria in Bolivia, which was 4.07 days [20], which is similar as in Shanxi Province. A study performed in Iran also revealed that there may be striking differences in time interval between onset of symptoms and diagnosis because of the scarcity and varying quality of detection in malaria epidemics, and case management and epidemic preparedness and response must be improved [30]. Therefore, the most important activities before “1” is to ensure every infection entering the chain of “1-3-7” approach could obtain early detection and it should continue to be strengthened in Shanxi Province. Public awareness, diagnosis, capacity maintenance, and adequate supplies and equipment should be supplied. This could ultimately facilitate the further prompt diagnosis and treatment [12, 23].

The import cases especially from Africa have further expanded the scope of the impact in Shanxi Province. To solve this, a well-function surveillance system should be carefully managed and further strengthened for the increasing imported cases especially around Chinese Spring Festival [3, 5, 37, 38]. Moreover, public awareness through health education should be enhanced and more plentiful supplies and equipment need to be made available [39, 40]. The effective multisector cooperation and coordination mechanisms particularly for CDC and hospitals should be strengthened such as information exchange and sharing [10, 37]. Training should also be improved mainly focus on the surveillance, emergence response and case diagnosis and treatment to avoid from severe malaria or unnecessary death attributing to malaria [30, 41].

This study has limitations due to data availability and low malaria cases. Firstly, the malaria cases reported in Shanxi Province is relatively low. In general, the trend of increased cases by year is unclear, and the analysis may not reflect the reality. Secondly, according to the literature [6, 27, 31], because of there is no data about treatment time, whether the delay affect the patient's treatment should be included in this study.

## Conclusions

This study found that long time interval occurred from fever onset to diagnosis of malaria in Shanxi Province. The work before “1” needs to be strengthened. This finding provides a reference for the development of malaria prevention strategies in Shanxi Province and other provinces or regions with similar elimination process.

## Data Availability

Data is available upon reasonable request to the corresponding author.

## Abbreviations

IDIRMS: National Infectious Diseases Information Reporting Management System
NMISM: National Malaria Information System Management
CDC: Center for Disease Control and Prevention
PCR: polymerase chain reaction
IQR: interquartile range
WHO: World Health Organization
